# Effects of factors of self-regulation vs. factors of external regulation of learning in self-regulated study

**DOI:** 10.3389/fpsyg.2022.968733

**Published:** 2022-08-04

**Authors:** Mónica Pachón-Basallo, Jesús de la Fuente, María C. González-Torres, José Manuel Martínez-Vicente, Francisco J. Peralta-Sánchez, Manuel M. Vera-Martínez

**Affiliations:** ^1^School of Education and Psychology, University of Navarra, Pamplona, Spain; ^2^School of Psychology, University of Almería, Almería, Spain; ^3^School of Psychology, University of Granada, Granada, Spain

**Keywords:** SRL vs. ERL Theory, self-regulation learning, external regulation learning, self-regulated study, metacognition

## Abstract

Since the mid-20th century, the study of Self-Regulated Learning (SRL) has aimed to identify the distinctive characteristics that enable individuals to acquire new knowledge and skills under their control. The theory of Internal Self-Regulation vs. External-Regulation in Learning (SRL vs. ERL; 2017) has postulated that a large number of self-regulatory variables are mediated by regulated/non-regulated or dysregulated features of the context. After signing their informed consent, a total of 616 university students completed validated instruments of SRL vs. ERL, behavioral regulation (SRB), regulatory teaching (RT), and metacognitive study control strategies (SRS). Using an ex-post facto design and correlation, regression, structural equation model and mediation analyses, the present research aimed to establish multicausal predictive relationships among the analyzed variables. Results indicated positive predictive effects between the external regulation variables on the self-regulation variables in learning [regulation (SRL)/non-regulation (NRL)/dysregulation (DRL)]; as well as positive predictive effects between SRL on SRB, RT and metacognitive SRS. Additionally, external regulation (ERL) not only predicted but mediated numerous relations among the variables studied. Other findings and important considerations for future research in the field of self-regulation are discussed.

## Introduction

In European countries such as for example Spain, Switzerland, France, Italy and Germany, the average adult will have been immersed in the formal education system for more than 15 years of their lives (The World Bank, 2021). Over that time, not only will their skills and difficulties associated with learning itself become apparent, but individuals will also be exposed to a range of contexts that may or may not facilitate the acquisition of new knowledge.

Based on models from prior research, such as Biggs’ 3Ps model (Biggs, 2003), the self-regulated learning model (SRL; Zimmerman et al., 2017) and the Theory of Self-Determined Behavior (Ryan and Deci, 2017) it has been posited that *effective teaching* is teaching that builds a teaching-learning environment which encourages learners to be committed to their own learning. More recently, in the framework of *SRL vs. ERL Theory* (de la Fuente, 2017), which puts forward a comprehensive vision of behavioral self-regulation and external regulation in the course of learning, important results have started to be seen in this direction (de la Fuente et al., 2017, 2019, 2020a,b; Pachón-Basallo et al., 2021; de la Fuente, 2022). In relation to SRL, the theory envisages that a student’s levels of self-regulation (SRL) and contextual external regulation (ERL) are distinct but complementary variables that combine in varying proportion (high/medium/low) to predict different aspects of the behavior of university students and their academic results.

This study, seeks in particular to explain how contextual variables associated with hetero-regulatory perception (within the family, school and peers) are associated with different levels of behavioral self-regulation in learning, general behavioral self-regulation, the perception of RT and the use of metacognitive strategies before, during and after study behavior. This research seeks to provide significant empirical evidence in the field of self-regulation and external regulation in the processes of teaching and learning.

### Self-regulated vs. externally regulated learning

There has been much research in Educational Psychology into SRL. However, that research has tended to focus on the subject and although some account is taken of the role of context, context has been seen as more peripheral and incidental. In fact, rather than seeing context as a ‘theater’ in which SRL is performed, we need to scrutinize the relationships between the subject and their context in relationship to learning ‘with a magnifying glass’ in a more systematic fashion. It is necessary, in addition, to carry out that scrutiny on the basis of a specific theoretical model, such as the model proposed by de la Fuente (2017) and have available instruments that are suitable to evaluate the predictions generated by the model.

#### Self-regulated, non-regulated, and dysregulated learning

The pattern of behaviors that characterize student’s predisposition to organize their learning can be broken down as follows:

(1) Self-regulated learning: this topic has been central to research in the psychology of education and among the most investigated by researchers since the mid-20th century (Torrano and González-Torres, 2004). Its influence has extended to many disciplines and fields (Special education, personality, health, business). Interest was first sparked by the work of Banduras on self-regulation of behavior in the 1970s and 1980s. When his research started to be applied to understanding the process of learning, the term SRL was coined and became popular in the 1980s and 1990s (González-Torres and Tourón, 1992; Dinsmore et al., 2008). Starting with the Zimmerman’s (1989) work titled *Self-Regulated Learning and Academic Achievement: Theory, Research, and Practice* a significant volume of important research has been conducted up until today (Popa, 2015; Roth et al., 2016; Gambo and Shakir, 2021). SRL is a broad term, such that it is not straightforward to identify and determine its boundaries and key processes. Numerous SRL models and theories developed by researchers focus on the description of the characteristics or attributes of students who self-regulate their learning processes (Roces and González-Torres, 1998; Puustinen and Pulkkinen, 2001; Panadero, 2017).

According to Zimmerman (1988), what characterizes students who self-regulate their learning is their active involvement in the regulation of three dimensions of learning: cognition, motivation and observable behavior. Other authors, such as Corno (1994); Kuhl (2000), and Pintrich (2000), add the dimensions of context and volition, respectively.

In general, those studies emphasize the following characteristics which differentiate students who self-regulate their learning from those who do not (Gonzáles-Torres and Torrano, 2008):

(a) Metacognitively and cognitively: They plan, monitor and direct their mental processes in order to achieve their aims (metacognition); they are aware of and use different cognitive strategies to acquire, develop and recover information.
(b) In terms of motivation, they are capable of generating, monitoring and modifying their motivational beliefs (for example: goals and expectations of self-efficacy) and their emotions to adapt them to the demands of a given task and a given learning situation.
(c) In terms of behavior, they are capable of creating and structuring environments that are conducive to learning (finding a suitable place to study, asking for help from teachers and classmates when they need it (help-seeking).
(d) In terms of context, whenever possible they join with the teacher in the selection and control of matters concerning tasks, the organization of classes, etc.
(e) In terms of volition, they are capable of creating and following habits that enable them to maintain their concentration, application and task persistence despite internal and external distractions.

One of the best known and accepted models, from a sociocognitive perspective, is the one proposed by Zimmerman (1989, 2015) which describes the different processes that are conducive to self-regulation of learning in three cyclical phases:

(A) Phase One, *preparation or planning*, takes place before the attempted learning starts. Its important elements are: goal setting, analysis of the tasks to be performed and the selection of the resources and strategies that will be used to achieve the goals set. In this phase, it is key to activate interest and beliefs in self-efficacy. To that end, specific, proximate and challenging goals are more effective than diffuse, delayed or easy goals to task motivation and good performance (Bandura and Schunk, 1981).
(B) Phase Two, *performance/control*, concerns performance, continuous monitoring and adjustment exercised by the subject during the task (maintenance of attention, observing, overseeing and monitoring progress (self-monitoring), self-instruction for the development of information, monitoring time and degree of application, mood, etc.).
(C) Phase Three, *final self-assessment*, comes after the performance phase and involves self-assessment of what has been achieved. Here, the subject reflects on what they have learned, on the level of performance reached in relation to the goals set, on the reasons for any successes or failures (causal attribution), evaluates their emotional reactions and degree of satisfaction, thinks about where and how to transfer what they have learned to other situations, and tries to identify errors so as to do better in future self-regulation cycles to address other tasks (Brainerd et al., 1989; Zimmerman et al., 2017).

As we can see, a student who adequately regulates their learning will demonstrate expertise in the process and the different phases described above, which are substantially the phases recognized by all models of SRL. However, we can place many students who behave in an unregulated manner or whose behavior is even dysregulated, at different points along a regulatory ‘continuum’. SRL vs. ERL Theory (de la Fuente, 2017) identifies these other levels of behavioral regulation.

(2) *Non-regulated learning (NRL)*: NRL can be conceptually defined as a lack of proactivity or the absence of self-regulatory behaviors (SRB) in the process of learning. Conceptually, it is equivalent to what has been mentioned by Zimmerman and Labuhn (2012) and Cohen (2012) in relation to reactive methods during the planning and performance phases. In this case, the individual is at the mercy of the external regulatory system to determine how they should behave.
(3) *Dys-regulated learning (DRL)*: DRL is a negative level of proactivity, i.e., an approach that is active but inadequate to regulate the individual’s own learning behavior. As can be seen, this dysregulation can have ‘negative consequences’ in terms of maintaining self-esteem, because individuals avoid the effort involved in proactive self-regulation and use self-handicapping, procrastination strategies, increased cheating in the exam hall, psychological reactance or other disruptive behaviors that ultimately do not promote learning or good psychological and moral adjustment (Valle et al., 2007; Muntada, 2013; Kapoor and Kaufman, 2020; Kapoor et al., 2021; Pachón-Basallo et al., 2021; Bakhtiar and Hadwin, 2022; Navarro-Patón et al., 2022).

#### External regulation, external non-regulation, and external dys-regulation of learning

General SRB and SRL are somewhat context-dependent, as underlined by Bandura. There are notable cognitive-social models that underlie research in this field, such as Zimmerman (2000, 2008). However, research has focused more on the description of the characteristics of students who self-regulate their learning. Although there are many studies as to how self-regulation can be supported, there is still a need for further studies that explore in detail the role of context in different fields (academic, social, family) and different levels (e.g., from the key elements of RT in general to instructional models of specific learning strategies). Further empirical evidence from that line of enquiry is necessary in order to explore further subject-context relationships and the different interactions that arise that are also the subject matter of SRL vs. ERL Theory.

That theory proposes that just as the subject can present three levels of self-regulation (regulation of behavior/learning; non-regulation of behavior/learning and dysregulation of behavior/learning), there are also contexts that make self-regulation more likely to occur, do not promote self-regulation or tend to lead to dysregulation of the subject. SRL vs ERL Theory therefore, categorizes external regulation in three levels (de la Fuente, 2017) that may be experienced by students in function of different patterns of signals and behaviors in the academic and other contexts that they inhabit. Those proposed levels are explained below:

(1) *Externally regulated learning*: In relation to the environment, Zimmerman and Schunk (1989) have highlighted the importance of the links between autonomous functioning and the context, specifically in the functional relationship between conduct and the environment. They emphasized the role of methods of instruction such as modeling, verbal instruction and reinforcement. According to them, external contingencies gradually promote self-regulatory responses. The presence of effective models is key to promoting a person’s capacity to regulate their own learning (Zimmerman and Schunk, 1989; Nilson, 2013). The distinctive feature of this type of regulation is that the context promotes positive or adequate proactivity. Thus, a regulatory context provides numerous stimuli that promote SRB in students, before, during and after the studying/learning processes. Those stimuli arise from background (patterns, standards, limits, expectations of successful self-regulation, value attributed to self-regulation, etc.) and from contextual consequences (positive and negative contingencies that favor self-regulation, adaptation, etc.). It has been found that a regulatory context negatively predicts psychological reactance and positively predicts self-regulation and academic confidence (de la Fuente et al., 2021b; Pachón-Basallo et al., 2021).
(2) *Externally NRL (ENL):* this level is characterized by the absence of stimuli that promote SRB in students: there are no external signs or stimuli that make self-regulated or unregulated behavior more probable at the beginning, during or at the end of the subject’s behavior in a learning situation. In a non-regulatory context, which is neutral toward regulation, an individual may engage in at least a moderate level of SRB, because there are no features of the context to steer them either toward greater self-regulation or toward dysregulation of their behavior. An example of external deregulation in the classroom might be the absence of clear guidance from the teacher as to the use of mobile devices in class when it is known that indiscriminate use of such devices by students is associated with increased cyberbullying, cheating and poorer mental health (Smale et al., 2021).
(3) *Externally DRL (EDL)*: in this level, a student’s context actively promotes dysregulation of learning. That is, “non-positive, inadequate, or negative proactivity” is externally promoted. There are many external signs or stimuli that make dysregulation of behavior more likely, favoring active dysregulation at the beginning, during and at the end of the behavioral episode. In this type of context, the individual has to make a great effort to attempt to self-regulate their behavior (de la Fuente, 2017). An example of this low level of external regulation might be manifested in inadequate, neglectful parenting and the influence of peers in encouraging the individual to adopt risky, dangerous behaviors that are counterproductive in terms of behaviors of academic engagement etc. (Pinho et al., 2021; Pérez Posada and Londoño-Vásquez, 2015).

### Behavioral self-regulation and regulatory teaching

#### Self-regulatory behavior

The construct of behavioral self-regulation has been extensively researched since the end of the twentieth century in multiple scenarios. SRB is conceived as a meta-skill in which cognitive processing is under control rather than automatic, such that through self-monitoring, self-evaluation, self-reinforcement/feedback the individual is capable of planning, guiding and monitoring their behavior in a way that responds flexibly to changing circumstances (Kanfer, 1986, 1970; Miller and Brown, 1991; Brown, 1998; Miller and Rollnick, 2013).

Carver and Scheier (1998) speak of the *cybernetic cycle* of SRB characterized by four stages: *test, operate, test*, *and exit*. The authors explain that a subject’s current behavior undergoes a process in which the subject compares it with a desired target behavior and then operates/acts to adjust their behavior until they confirm that their level of performance is at the initial target level. When the answer at the test stage is positive, the subject moves to the exit stage and the cycle starts again. In summary, SRB is behavior that seeks to reduce the discrepancy between target (desirable) behaviors and actual behaviors. That requires the person to be capable of constant feeding back to themselves concerning the narrowing or widening of any gap and adjusting their efforts and strategies to achieve the target behavior.

Self-regulated behavior and a lack of SRB have been extensively linked to sports performance, driving behavior in traffic psychology and to the general notion of people’s lifestyle (Hennessy et al., 2011; Miller and Rollnick, 2013; Goffena and Horn, 2021). Many deficits of self-regulation have been linked specifically to risk behaviors such as substance abuse, impulsivity, procrastination, problem behaviors relating to food, etc. Also, from a social perspective, deficit of self-regulation has been linked to crime, teenage pregnancies, STIs, gambling addiction, domestic violence, etc. (Miller and Brown, 1991; Baumeister and Heatherton, 1996; Brown et al., 1999; Garzón-Umerenkova et al., 2018; Watson-Brown et al., 2021); Baumeister and Heatherton (1996) indicate that deficits in or lack of the capacity to self-regulate may be due to failures of self-control, of realistic goal selection, to the absence of skills compatible with the target behaviors, etc. Karniol and Miller (1983) in turn indicate that such failures of self-regulation may be preceded by changes in attention to different types of reward. Self-regulation requires the selection of long-term reward in preference to immediate reward that at any given moment could appear more attractive (Duckworth et al., 2013).

#### Regulatory teaching

Entwistle and Peterson (2004) suggest that effective teaching takes place when a teacher creates a classroom atmosphere in which students commit to processing content and take responsibility for their own learning. In that connection, RT (de la Fuente et al., 2012) has been defined as a contextual variable in which teaching externally promotes and favors SRL in students (Yerdelen and Sungur, 2019). Empirical research identifies high-quality teachers as those who positively influence the commitment of their students to learning activities and to their own learning performance (including social skills, academic performance and self-regulation; Goe et al., 2008; de la Fuente et al., 2012).

Instruction is an intentional process, such that it is the educator’s self-regulation of their teaching process that allows them to take effective decisions in the different phases of the educative process (Biggs, 2001, 2003). Various mediating factors in students’ self-regulation of their learning and performance depend on the teacher as *adaptive expert* (Hammerness et al., 2005). The determination of clear teaching goals derived from an assessment of needs, the organization of content and planned activities carried out in the classroom to foment deep processing and evaluate it (Roehrig and Christesen, 2010). That is why the perception that students have of how their teachers teach is fundamental. Recent research has shown that variables of the learning environment perceived in the classroom are good predictors of self-regulation of learning by students and their self-perception (Biggs, 2001; Zimmerman, 2002; Pintrich, 2004; Schunk, 2005; Monereo, 2007; Schuitema et al., 2012).

The perception that students have of their educational experience is similarly a widely studied variable in multiple contexts. Regulatory learning, first, facilitates students’ monitoring of their own academic performance and their satisfaction with learning. There is evidence that the gradual increase of internal and external regulation predicts increased academic confidence and decreased procrastination behaviors (de la Fuente, 2017; Putwain and Pescod, 2018; de la Fuente et al., 2021a); Baherimoghadam et al. (2021) found that even in online teaching processes there is a significant relationship moderated between perceptions of self-efficacy (i.e., the beliefs that students have about their own capacity to organize and execute the courses of action required to achieve specific outcomes (Bandura, 1986) and the level of satisfaction with the learning process.

It is important to note that educational institutions are themselves extremely interested in the perception that students have of the teaching that they receive. In fact, student satisfaction with the teaching-learning process is used as a measure of educational quality (Booth et al., 1999; Bobe and Cooper, 2019). A recent meta-analysis by Caskurlu et al. (2020) indicates that numerous studies have shown that the presence of teaching staff significantly predicts student satisfaction. Anderson et al. (2019), define RT as the design, direction and facilitation of social and cognitive processes that the teacher offers with the aim of obtaining learning outcomes that are significant to the student. Continuous feedback and direction, promotion of motivation, interest and commitment are essential components of RT.

In the aggregate, whilst student satisfaction with the teaching-learning process is generally associated with different factors such as teaching methods, course content, the learning environment, relationships with administrative departments and the learning community (Holdfor and Patkar, 2003), the research carried out by Wu et al. (2015) revealed that it is course content that best predicts that satisfaction. They placed particular emphasis on the planning of course content that matches the needs of students. In addition, those authors found that satisfaction with learning predicts the intention to continue to participate in future formal educational processes.

By way of summary, it can be assumed that adequate design and implementation by teachers of the teaching-learning process will facilitate students seeing learning as theirs, regulating it procedurally and attitudinally (knowing how, wanting to know and doing) and not just conceptually (knowing) (de la Fuente et al., 2014).

### Metacognitive study control strategies

As has been said, students can regulate three important dimensions of learning: cognition, motivation/emotion and apparent behavior, as well as context factors. To do so, they use different kinds of strategies: cognitive, metacognitive and support (Dansereau, 1985; González-Torres, 1997; González-Pienda et al., 1998; Pintrich, 2004).

Cognitive strategies include study habits and different resources that assist in the process of comprehension, codification, and recall of information that Weinstein and Mayer (1986) break down in their well-known classification as: strategies of rehearsal, elaboration and organization. Those strategies and so-called *support strategies* (Dansereau, 1985), which indirectly assist cognitive processing by creating a psychological climate that is conducive to the maintenance of concentration and motivation, are not in themselves sufficient to ensure good learning. What really distinguishes students who learn well from those who learn badly is not just, as Nisbet and Shucksmith (2017) would say, the possession of a certain level of intelligence or a series of effective study methods or techniques, but the capacity to capture the demands of the task and monitor the learning situation and that is called metacognition. So-called metacognitive or secondary strategies (Dansereau, 1985) are at the heart of SRL, they are key to it. A student’s learning will be poor if they do not know and they are not shown how to plan, monitor and direct their own mental and psychological processes to adjust those processes to the demands of the task (González-Torres, 1997).

Metacognition, a term introduced by Flavell (1987) includes two dimensions: (a) metacognitive knowledge which includes being aware of the personal variables of the task and the strategies that affect performance on a task and (b) metacognition as self-monitoring. Metacognition in this regulatory dimension includes three principal ingredients: planning, monitoring and evaluation of what has been achieved. A student who monitors their learning process is a student who asks themselves questions such as: what is the purpose of the task? What strategies am I going to use? Am I achieving what I set out to do? What have I achieved and how can I improve? That reflective attitude before, during and at the end of the learning process makes students expert strategic thinkers or learners (Flavell, 1987; Ertmer and Newby, 1996; González-Torres, 1997).

There has been considerable research into the metacognitive and behavioral strategies that students use during a specific study activity (Lanza and Sánchez, 2014; Campano et al., 2017). The *Strategies for Control of Study Questionnaire* by Hernández and García (1995) assesses metacognitive strategies in three dimensions or factors: planning, oversight and review.

*Planning* includes behaviors in which the activities to be performed are organized in specific orders, including the time allowed for each in order to meet a study goal. This sub-category also includes, as González-Torres and Tourón (1992) mention, subdivision of tasks, the generation of questions in the face of new material, creating hypotheses, etc. *Oversight* includes review of what has been studied, including aspects that could be improved, i.e., the efforts that a student makes to observe their own behavior (Rodríguez, 2009). Finally, the *factor of review* includes the search for help from third parties when it is required and the self-evaluation of everything done over the period of study. In the *evaluation* phase, as Rodríguez (2009) indicates, the subject engages in reflection concerning the study process and their own learning, feeding back into the choice of study methods to achieve their next objectives.

It is important to note that there have been studies looking at whether there are or are not variables that could affect the use of those strategies such as might be age, academic year (Aluja-Fabregat and Blanch, 2004; Inglés et al., 2013; Campano et al., 2017). In the study conducted by Inglés et al. (2013), it was found that the use of learning strategies stagnates as students reach later academic years. The authors explain that this may because around fifteen years of age, students have already settled on strategies that they consider effective and tend to reuse them. However, in university populations it has been found that there are significant differences between different academic levels and the use of metacognitive strategies. Students who are approaching the end of their degrees are those who most use such strategies (Martínez-Fernández, 2007).

Elsewhere, a positive relationship has been observed between the use of metacognitive strategies and academic performance (Caso-Niebla and Hernández-Guzmán, 2007; Young and Fry, 2012). In the research undertaken by Caso-Niebla and Hernández-Guzmán (2007) in a population of more than 1500 students, they were able to determine that women tend to make the greatest use of study strategies and skills. In addition, the evidence also indicates (Rodríguez, 2009) that the use of control strategies in study is related to the orientation/motivation of a student toward learning. Motivational variables may influence not only performance but also the quality with which storage, processing and use of information operations that form part of the process of study are performed (González-Torres and Tourón, 1992; González-Torres, 1997). Thus, as shown by McCombs and Marzano (1990), the characteristics of students who regulate their learning are a combination of *Will* and *Skill*.

In other significant research, it has been found that pro-social behavior significantly positively predicts the use of study strategies such as the selection of the principal ideas to be studied, the search for help, self-evaluation and exam preparation, among others (Inglés et al., 2013). Finally, Lanza and Sánchez (2014) were able to conclude that no significant differences in terms of the use of learning strategies in relation to the parental support in the conducting of study tasks are found. However, the variable did impact student’s organization and self-regulation.

### Objectives and hypothesis

Despite the extensive evidence mentioned, there is still scant information concerning predictive and mediating variables relative to metacognitive regulatory strategies in the course of study, specifically concerning the effects of students’ contexts. Consequently, the objective of this study was to determine those predictive relationships. The following hypotheses were postulated:

#### Hypotheses of association

(1) We expected to find a positive correlation between learning regulation variables of the subject and their context (SRL/ERL), and variables of general SRB, RT, and self-regulated study behavior (SRS). We also expected to find a negative correlation between the variables of non-regulation and dysregulation of the individual and their context (NRL/ENL and DRL/EDL) with those same variables (SRL/ERL/SRB/RT/SRS).
(2) We expected to find a positive correlation between corresponding internal and external levels of regulation of learning: (regulated) SRL with ERL; (non-regulated) NRL with ENL, and (dysregulated) DRL with EDL.

#### Predictive linear hypotheses

(3) It was expected that large part of the variation in the variables of SRB, RT, and SRS would be explained by variables of both subject and context (SRL/NRL/DRL-ERL/ENL/EDL). And that SRL would positively predict RT and general SRB. Together, SRL and RT would positively predict SRS. We also expected that SRB would be negatively predicted by both NRL/ENL and DRL/EDL.
(4) Each level of external regulation of learning will predict the same level of self-regulation: ERL will predict SRL; ENL will predict NRL; EDL will predict DRL. In complementary fashion, both internal and external non-regulation will positively predict internal and external dysregulation of learning, that is: ENL predicts EDL and NRL predicts DRL.
(5) We expected to find significant models of mediation in which especially SRL mediates the relationship between contextual variables and other personal variables such as NRL and DRL.

## Materials and methods

### Participants

A total of 616 students from different universities voluntarily participated in this research. The sample was composed of students particularly in the fields of psychology, education and other social sciences. Of the total, 68.9% were women and 31.1% were men. The age range was 17–34 and the mean age was 22.19 years (SD = 3.19). The sample was incidental rather than probabilistic because the sample could not be randomized. The students voluntarily completed self-reports in a learning context (i.e, classes of different university subjects). Participation was anonymous and voluntary. The questionnaires were completed online.

### Instruments

#### Self-regulation vs. external regulation of learning

That questionnaire (de la Fuente, 2020), is structured in six sub-scales, with six items each that assess behaviors related to learning, both in the person and their context: (1) SRL (“I am aware of my learning and academic performance needs.”). (2) ERL (“The context in which I live (family, setting, friends) helps me to plan my behavior, through learning, study and performance goals and objectives.”). (3) Internally NRL (“I don’t need to make any decisions to make changes in my learning and study behaviors.”). (4) Externally NRL (“In the context that I live in (family, environment, friends) we rarely talk about my behavior and what I need to do to improve my learning, study and academic performance.”). (5) Internally dysregulated learning (DRL) (“I take decisions to have the most fun, even at the expense of my learning, study and performance aims.”), and (6) EDL (“The context in which I live (family, environment, friends) encourages me to focus on taking decisions to enjoy the moment and to postpone learning and study decisions that are important for me.”). Its confirmatory factorial structure is consistent in this sample (Chi Square = 1650,992, df = 579, *p <* 0.001; Ch/df = 2.851; RMSR = 0.05; IFI = 0.91; TLI = 0.90; CFI = 0.91; RMSEA = 0.05). The internal reliability figure for the instrument was good (α = 0.87; ω = 0.84).

#### Self-regulated behavior

This variable was measured using the abbreviated version of the Self-Regulation Questionnaire (SRQ; Miller and Brown, 1991). That instrument has been validated in Spanish samples (Pichardo et al., 2018) and has acceptable validity and reliability values comparable to the English version. The abbreviated SRQ is composed of four factors: (1) Goal-setting (“Once I have a goal, I can usually plan how to achieve it.”). (2) Perseverance (“I am easily distracted from my plans.”). (3) Decision-making (“When it comes to deciding on a change, I feel overwhelmed by the decisions.”), and (4) Learning from mistakes (“Usually, once I’ve made a mistake once, I learn from it.”). It has 17 items (all with saturation >0.40) with a consistent confirmatory factorial structure (Chi-square = 595.052, df = 113, *p* < 0.001; Ch/df = 5.26; SRMR = 0.07; CFI = 0.97, RFI = 0.96, IFI = 0.97, TLI = 0.96, GFI = 0.97, RMSEA = 0.08). Internal consistency was acceptable for the total of items in the questionnaire in this sample (α = 0.84; ω = 0.84).

#### Regulatory teaching

The abbreviated Interactive Evaluation of the Teaching-Learning Process Scales (EIPEA, in Spanish) (de la Fuente et al., 2012), were used to assess students’ perception of how they see the provision of teaching, their SRL on their course and their satisfaction with both. The instrument has three dimensions: (1) RT, which incorporates the factors of evaluation, preparation, satisfaction with teaching (“When we are learning, the teacher helps us to set clear, realistic learning goals.”), (2) SRL, which refers to factors of planning, significant learning and the use of study techniques (“Before starting a learning activity or task, I usually consider what I need to know and how long I have to give to it.”). (3) Outcome, comprising two factors associated with the final product of the learning process: satisfaction with learning and significant learning (“I have learned the goals set well enough.”). In this abbreviated version, 37 items were used and the confirmatory factorial structure of the scale was acceptable (χ^2^ = 2260,907, df = 492, *p* < 0.001; Ch/df = 4.59; SRMR = 0.05; CFI = 0.84, NFI = 0.85, RFI = 0.802, TLI = 0.83, NNFI = 0.80, RMSEA = 0.07) and the internal reliability value of the instrument is excellent (α = 0.94; ω = 0.96).

#### The regulatory strategies in study questionnaire (SRS)

This questionnaire (Hernández and García, 1995), has a structure with 17 items and three factors, which are planning, oversight and review. Completion of the questionnaire requires students to indicate the extent to which they agree with the strategies used, both at the outset (“Before starting to study, I usually think about what I need to study, what activities I have to do or how much work or time studying is going to take.”), during (“If there is something I don’t understand or don’t know how to do, I try not to move forward until I have understood it.”) and at the end of periods of study (“When I have studied a topic and it’s been a while, I try to go back over it or refresh it in my memory before a test or exam.”). There are five possible responses from “1. If you never usually do what the sentence says” to “5. If you normally do it a lot or always.” The confirmatory factorial structure for the scale is consistent in this sample (χ^2^ = 462,242, df = 116, *p <* 0.001; Ch/df = 3.98; SRMR = 0.05; CFI = 0.90, GFI = 0.91, AGFI = 0.90, RMSEA = 0.07) and the internal validity value is good (α = 0.86; ω = 0.85).

### Procedure

The participants in this research were invited to participate in the study voluntarily. After giving informed consent, they completed the scales using an online platform that ensured the anonymity of their responses. Students registered on the platform at this url: http://www.inetas.net. That tool provides assessment and intervention in a self-help system for the university students and their teachers. The R&D project was approved by the Ethics Committee of the University of Navarre (ref. 2018.170). Compliance with the ethical principles of psychology was ensured (de la Fuente et al., 2015).

### Data analysis

As a preliminary step, we confirmed the normal distribution of the sample by the Kolmogorov–Smirnoff test for dependent variables (Lohr, 2010). We also used the Hoelter index to determine the adequacy of the size of the sample (Tabachnick and Fidell, 2001). Analyses of linearity and atypical values, missing cases and critical multivariate normality values were in addition performed. The values recommended for the multivariate kurtosis ratio or Mardia’s coefficient were below 70 (Mardia, 1970).

For the association hypotheses (1 and 2), bivariate Pearson correlations were performed. For the prediction hypotheses (3, 4. and 5), linear regression analyses were used, and it was confirmed through remainder analysis that the data were adequately compliant with the assumptions of the linear regression model. Subsequently, predictive structural equation modeling (SEM) was performed (Weston and Gore, 2006; Kline, 2016). For that purpose, we followed the recommendations of Hu and Bentler (1999) and Hair et al. (2010), in which a model is adequately adjusted to the observed data when the ratio of chi-square to the degrees of freedom is below five, RMSEA and SRMR are <0.08 and NNFI (non-normal fit index), IFI and CFI are >0.90 for an acceptable model (Jöreskog and Sörbom, 1998). We used maximum likelihood of robust standard errors (MLR estimation) for estimation given its applicability to non-normal data. Participants with missing data were included in the estimation of the model using full information maximum likelihood (FIML) to avoid any distortion of analysis from missing values (Enders and Bandalos, 2001). Reliability of the dimensions of the model, of the overall structure and each of the factorial structures proposed was also examined by calculation of Cronbach’s alpha (Quero-Virla, 2010). In addition, account was taken of the recommendations of Keith (2019) for cutoff criteria for direct and indirect effects: <0.05 deemed to be too small to be significant, above 0.05 is small but significant, effects above 0.10 are moderate and above 0.25 are large effects.

The computer programs used to conduct this analysis were SPSS 26.0 (IBM Corp, 2019) for reliability and AMOS v. 23.0 (Arbuckle, 2014) for confirmatory factorial analysis and SEM.

## Results

### Prior analyses

The results of the Kolmogorov–Smirnov test (*p* < 0.001) and the Shapiro–Wilk test (*p* < 0.001) were significant, such that analyses appropriate for non-parametric samples were performed. In general terms, regulatory variables (SRB; SRL; ERL; RT; RS) had means and medians higher than those for non-regulation or dysregulation, both internal and external. In addition, those variables showed negative asymmetry in which the values observed tended to be concentrated in the superior/higher segment of the relevant scales (see Table 1).

**TABLE 1:** Preliminary analyses.

	SRB	SRL	NRL	DRL	ERL	ENL	EDL	RT	SRS
Mean	3.333	3.874	2.654	2.409	3.670	2.604	2.353	3.781	3.852
Mean standard error	0.024	0.029	0.028	0.035	0.039	0.037	0.039	0.026	0.023
Median	3.350	4.000	2.666	2.333	3.833	2.666	2.166	3.831	3.865
IQR	0.80	1.00	1.00	1.17	1.33	1.17	1.50	0.80	0.83
Mode	3.23	4.00	2.50	2.33	5.00	3.00	1.00	3.00	5.00
Standard deviation	0.605	0.743	0.711	0.874	0.979	0.933	0.981	0.645	0.588
Asymmetry	–0.239	–0.422	0.219	0.361	–0.530	0.153	0.388	–0.651	–0.455
Standard asymmetry error	0.099	0.098	0.098	0.099	0.098	0.098	0.098	0.098	0.099
Kurtosis	0.058	–0.311	0.107	–0.227	–0.244	–0.384	–0.535	0.778	0.248
Standard kurtosis error	0.197	0.197	0.197	0.197	0.197	0.197	0.197	0.197	0.197
Range	3.53	3.33	4.00	4.50	4.17	4.17	4.17	4.00	3.50
Minimum	1.38	1.67	1.00	0.50	0.83	0.83	0.83	1.00	1.50
Maximum	4.90	5.00	5.00	5.00	5.00	5.00	5.00	5.00	5.00

SRB, self-regulated behavior; SRL, self-regulated learning; NRL, non-regulated learning; DRL, dysregulated learning; ERL, externally regulated learning; ENL, externally non-regulated learning; EDL, externally dysregulated learning; RT, regulatory teaching; SRS, self-regulated study; IQR, interquartile range.

### Linear association

The internal and external variables of regulation of learning (SRL/ERL) were significantly positively correlated with each other. In addition, the different levels of both internal and contextual non-regulation and dysregulation were significantly positively correlated (NRL/ENL, DRL/EDL). Levels of SRL and ERL were significantly negatively correlated with levels of internal and external non-regulation and dysregulation (NRL/ENL, DRL/EDL).

Following the same trend, and relative to the variables of SRB and RT, the results showed significant positive correlations between those two variables and SRL and ERL. SRB and RT were significantly negatively correlated with NRL/ENL and DRL/EDL.

Finally, SRS tended to be significantly positively correlated with RT, SRB and with SRL and ERL. Conversely, SRS was significantly negatively correlated with DRL/EDL and NRL/ENL (see Table 2).

**TABLE 2:** Bivariate correlations between SRL vs. ERL variables and SRB, RT, and RS (*n* = 616).

	SRL	NRL	DRL	ERL	ENL	EDL	SRB	RT
SRL								
NRL	–0.399***							
DRL	–0.264***	0.658***						
ERL	0.513***	–0.219***	–0.159***					
ENL	–0.263***	0.512***	0.532***	–0.292***				
EDL	–0.161***	0.469***	0.638***	–0.153***	0.650***			
SRB	0.455***	–0.311***	–0.289***	0.312***	–0.203***	–0.135**		
RT	0.544***	–0.267***	–0.218***	0.351***	–0.220***	–0.181***	0.412***	
SRS	0.548***	–0.219***	–0.190***	0.378***	–0.200***	–0.156***	0.375***	0.585***

SRL, self-regulated learning; NRL, non-regulated learning; DRL, dysregulated learning; ERL, externally regulated learning; ENL, externally non-regulated learning; EDL, externally dysregulated learning; SRB, self-regulated behavior; RT, regulatory teaching; SRS, self-regulated study.

**p < 0.05, ***p < 0.001.

### Linear and structural prediction

#### Linear regression

Table 3 shows the linear regressions for different variables (in bold), relative to different groups of independent variables. Almost half (47%) of the variability of SRB was explained by SRL and ERL/NRL and DRL/EDL (but not ENL) [*F*(8,608 = 94.088, *p <* 0.001)]. SRL and ENL were the most significant subject variables.

**TABLE 3:** Standardized simple linear regression coefficients (*n* = 616).

	β	*T*	Significance	*R* ^2^
**(1) SRB**				0.476
SRL	0.544	15.666	0.000	
NRL	–0.155	–3.679	0.000	
DRL	–0.172	–3.672	0.000	
ERL	0.099	2.829	0.005	
ENL	0.033	0.767	0.443	
EDL	0.101	2.300	0.022	
**(2) RT**				0.523
SRL	0.597	18.012	0.000	
NRL	–0.071	–1.776	0.076	
DRL	–0.072	–1.610	0.108	
ERL	0.150	4.502	0.000	
ENL	0.015	0.375	0.707	
EDL	–0.014	–0.328	0.743	
**(3) SRS**				0.495
SRL	0.629	18.449	0.000	
NRL	0.011	0.275	0.784	
DRL	0.003	0.063	0.950	
ERL	0.122	3.564	0.000	
ENL	0.008	0.192	0.848	
EDL	–0.089	–2.067	0.039	
**(4) SRS**				0.595
SRB	0.437	14.436	0.000	
**(5) SRL**				0.537
ERL	0.227	7.301	0.000	
RT	0.598	19.236	0.000	
**(6) NRL**				0.286
ENL	0.535	15.686	0.000	
**(7) DRL**				0.518
ERL	–0.015	–0.511	0.610	
NRL	0.406	12.222	0.000	
EDL	0.418	12.618	0.000	

SRB, self-regulated behavior; SRL, self-regulated learning; NRL, non-regulated learning; DRL, dysregulated learning; ERL, externally regulated learning; ENL, externally non-regulated learning; EDL, externally dysregulated learning; RT, regulatory teaching; SRS, self-regulated study.

In relation to RT and SRS, it was observed that for both variables, approximately half of the variability (52 and 49%, respectively) was explained by the variables of SRL and ENL. With a lesser degree of significance (*p* < 0.05), the variability of SRS was partially negatively explained by EDL [*F*(6, 608) = 101.257, *p* < 0.001)]; similarly, SRB explained more than half of the variation of EDL [*F*(2, 613) = 453.028, *p* < 0.001)].

Within SRL and ERL, it was found that ERL and RT explained approximately 53% of the variability of SRL [*F*(2, 613) = 354.817, *p* < 0.001)]. In turn, around 28% of NRL was explained by ENL [*F*(1, 614) = 264.047, *p* < 0.001)]. The same pattern was found with dysregulation variables: EDL together with NRL explained more than 50% of the variability of DRL [*F*(3, 600) = 214.772, *p* < 0.001)].

#### Structural model

**FIGURE 1:**
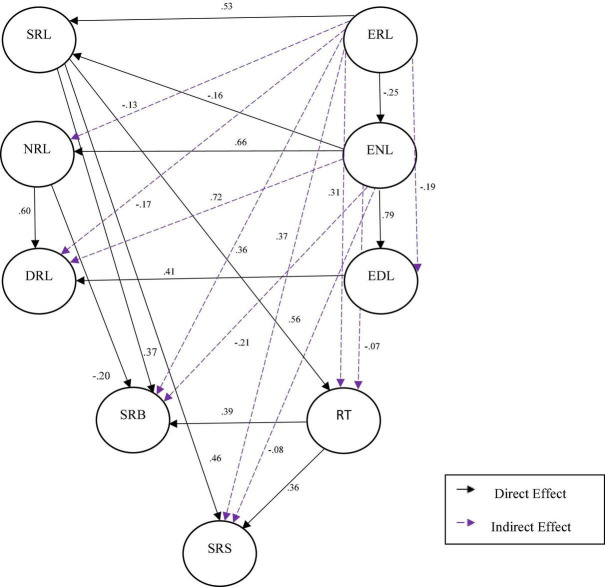
Predictive structural equation model: direct and mediational effects. SRL, self-regulated learning; NRL, non-regulated learning; DRL, dysregulated learning; ERL, externally regulated learning; ENL, externally non-regulated learning; EDL, externally dysregulated learning; SRB, self-regulated behavior; RT, regulatory teaching; SRS, self-regulated study.

Two models of structural equations were tested: **Model 1** tested the prediction for the relationship between the external factors of ERL, ENL and EDL with the internal factors of SRL, NRL, and DRL; and for the relationship between RT and SRB, SRL and ERL and the predictive effect of SRL, RT, and EDL in relation to SRS. **Model 2** generated the closest ratios and prediction of internal variables by external variables was maintained. We tested SRL as a predictor of RT, SRS and SRB. We also assessed the predictive effects of NRL for SRB; of SRB and RT for SRS; of RT for SRS; and, ENL for SRL (see Table 4; Figure 1).

**TABLE 4:** Models of structural linear results for the variables.

Model	χ^2^	DF	CH/df	SRMR	*p*<	IFI	TLI	CFI	RMSEA	HOELT 0.05	HOELT 0.01
1	2805.967	978	2,869	0.076	0,001	0,876	0,869	0,876	0,055	231	238
2	2224.142	905	2,446	0.065	0,001	0,908	0,911	0,909	0,048	272	270

##### Direct and indirect effects

In relation to the direct predictive effects or internal and external self-regulation, the results showed that SRL positively predicted SRB, RT, and SRS. In turn, NRL had a significant positive direct effect on DRL and a negative predictive effect for SRB. In relation to external factors, ERL had an important predictive effect for SRL and a negative predictive effect for ENL. ENL had significant positive predictive effects for NRL and EDL; conversely, ENL was negatively predictive for SRL. EDL had a positive predictive effect for DRL. Finally, RT had a positive predictive effect for SRB and SRS (see Table 5; Figure 1).

**TABLE 5:** Total, indirect, and direct effects of the variables in this study and 95% bootstrap confidence intervals (CI).

Predictive variable	Criterion variable	Total effect	CI (95%)	Direct effect	CI 95%	Indirect effect	CI 95%	Results, effects
SRL →	SRB	0.595	(0.498, 0.660)	0.374	(0.226, 0.487)	0.217	(0.145, 0.298)	P.M.
	RT	0.562	(0.456, 0.639)	0.562	(0.456, 0.639)			D.O.
	SRS	0.663	(0.576, 0.735)	0.462	(0.333, 0.587)	0.201	(0.114, 0.281)	P.M.
NRL→	DRL	–0.596	(–0.506, 0.688)	–0.596	(0.506, 0.688)			D.O.
	SRB	–0.202	(–0.314, –0.104)	–0.202	(–0.314, –0.104)			D.O.
ERL→	SRL	0.566	(0.490, 0.640)	0.534	(0.451, 0.614)	0.032	(0.013, 0.068)	P.M.
	NRL	–0.165	(–0.257, –0.111)			–0.165	(–0.257, –0.111)	F.M.
	DRL	–0.179	(–0.265, –0.119)			–0.179	(–0.265, –0.119)	F.M.
	ENL	–0.249	(–0.361, –0.159)	–0.249	(–0.361, –0.159)			D.O.
	EDL	–0.197	(–0.278, –0.131)			–0.197	(–0.278, –0.131)	F.M.
	RT	0.318	(0.237, 0.388)			0.318	(0.237, 0.388)	F.M.
	SRB	0.368	(0.301, 0.434)			0.368	(0.301, 0.434)	F.M.
	SRS	0.375	(0.306, 0.439)			0.375	(0.306, 0.439)	F.M.
ENL→	SRL	–0.130	(–0.216, –0.058)	–0.130	(–0.216, –0.058)			D.O.
	NRL	0.664	(0.580, 0.733)	0.664	(0.580, –0.058)			D.O.
	DRL	0.720	(0.661, 0.781)			0.720	(0.661, 0.781)	F.M.
	EDL	0.791	(0.725, 0.854)	0.791	(0.725, 0.854)			D.O.
	RT	–0.073	(–0.122, –0.032)			–0.073	(–0.122, –0.032)	F.M.
	SRB	–0.211	(–0.307, –0.119)			–0.211	(–0.307, –0.119)	F.M.
	SRS	–0.086	(–0.149, –0.039)			–0.086	(–0.149, –0.039)	F.M.
EDL→	DRL	0.410	(0.313, 0.854)	0.410	(0.313, 0.503)			D.O.
RT→	SRB	0.387	(0.281, 0.505)	0.387	(0.281, 0.505)			D.O.
	SRS	0.357	(0.219, 0.487)	0.357	(0.219, 0.487)			D.O.

SRL, self-regulated learning; NRL, non-regulated learning; DRL, dysregulated learning; ERL, externally regulated learning; ENL, externally non-regulated learning; EDL, externally dysregulated learning; SRB, self-regulated behavior; RT, regulatory teaching; SRS, self-regulated study; P.M., partial mediation; F.M., full mediation; D.O., direct only; CI, confidence interval. Bootstrapping sample size = 200.

In relation to indirect predictive effects (see Table 5), it was found that SRL had positive indirect effects for SRB and SRS. In relation to contextual variables, ERL was the variable with the greatest number of indirect effects on other variables: it had a negative effect for NRL, DRL, EDL, SRB, RT and SRS. ENL had an indirect positive effect for DRL and conversely showed a negative indirect effect for SRB.

##### Mediation relationships

Taking into account the direct and indirect effects described and through an analysis of the total effects (Table 5), we found eight full simple mediations and two full multiple mediations, i.e., relationships in which the predictive effect was not direct but rather mediated by other variables. They are described below:

Self-regulated learning mediated the relationship between: (1) ERL and SRS; (2) ENL and SRS; (3) ENL and SRB; (4) ERL and SRB; (5) ERL and RT; and (6) ENL and RT. ENL mediated the relationship between: (7) ERL and EDL; and (8) ERL and NRL.

The following were characterized by multiple mediation: the relationship between ERL and DRL, which was mediated by the indirect effects of ERL and of EDL as well as NRL. The relationship between ENL and DRL was mediated by the direct effects of the relationships between ENL and NRL and EDL.

Three partial mediations were found: (1) **RT** partially mediated the relationship between SRL and SRB and (2) and between SRL and SRS. Finally, we found evidence that the predictive relationship of ERL relative to SRL was partially mediated by **ENL**.

## Discussion

All students can learn to regulate their learning, because the capacity for self-regulation is not a personality type or trait that a person cannot control, but rather something modifiable and capable of change that can be improved with, among other things, the help of an appropriate teaching environment (Roces and González-Torres, 1998; Bakhtiar and Hadwin, 2022). The findings of this study are consistent with other prior studies which highlight the importance of context in predicting regulatory behavior among students; they are significant not only in the field of education but in all contexts in which students need to exercise control over their own behavior. Thus, although the SRL vs. ERL model arose in the context of SRL, it has at least in part shown itself to be a miore generally applicable model (SR vs. ER) which can be used to assess self-regulation and external regulation in other contexts, such as health (de la Fuente, 2017, 2020, 2021; Hwang et al., 2021; Pachón-Basallo et al., 2021).

Thus, in relation to hypotheses 1 and 2, the results showed that SRL and ERL were positively correlated with RT, SRB and with SRS. Those variables (RT, SRB, and SRS), were significantly negatively correlated with NRL and ENL. That is consistent with the findings of earlier research and is evidence of the external validity of the theoretical construct previously put forward by de la Fuente (2017) based on earlier theories of self-regulation of behavior (Zimmerman, 1988; Zimmerman and Schunk, 1989; Zimmerman and Labuhn, 2012; Pachón-Basallo et al., 2021).

The results described in this study prove the interdependence identified by Bandura (1986) between contextual and personal variables. As well as those mentioned and in relation to hypotheses 3–5 (prediction and mediation), the following relationships are notable:

(a) External-regulation learning was significantly positively predictive of SRL; ENL predicted NRL, and EDL predicted DRL. As an additional finding, in this study we identified that perceptions of RT positively predicted SRB.
(b) Non-regulated learning and ENL predict DRL and EDL with significant predictive weights (0.60 and 0.79, respectively). The data indicate that personal dysregulatory behaviors, such as procrastination, psychological reactance, etc. and contextual factors such as inadequate family guidance, risky group behaviors, etc., can be predicted by the absence of norms or other clear aspects of context that could steer the behavior of students before, during and after performance. The same results have also been found in the field of health, where the absence of orientating stimuli positively predicted internal and external dysregulation in relation to health adjustment behaviors in university students. In addition and in relation to executive functioning, it has been found that non-regulatory and dysregulatory contexts are positively associated with executive dysfunction and problems with emotional regulation (Pachón-Basallo et al., 2021; de la Fuente, 2022).
(c) In a direct and interactive way, EDL had a significant positive effect on DRL (0.72), which was mediated by both NRL and EDL.
(d) SRL mediated the relationship between contextual variables such as ERL and ENL relative to SRS and SRB. It can be said that a regulatory context favors SRL (directly through ERL and indirectly and negatively through ENL, which in turn favors SRB, SRS and the perception of RT.

Those findings can help to answer the question why some students are not always satisfied with their own capacity for self-regulation despite recognizing the nexus between regulating themselves and improved academic results (Koenig and Guertler, 2021). In addition, the findings can complement the analysis conducted by Baumeister and Heatherton (1996) of failure in SRB, which focused mainly on personal factors (such as goal selection, self-monitoring, manifestation of inappropriate behaviors and stress/fatigue). The principal contribution of this research concerns the role of context, and the data show that context has a considerable predictive weight for student behavior. In fact, the perception that a student has of their immediate context supports or does not support certain regulatory decisions, will facilitate the use that the student will tend to make of metacognitive strategies in the study process and the specific metacognitive strategies that the student will tend to use in that process. Our results confirm what was found by Baumeister and Heatherton (1996) concerning the considerable influence that culture can have when teaching individuals about the circumstances in which loss or release of control is or is not appropriate. These findings first highlight the need for the community to act to prevent and reduce risky behaviors in young people in many contexts, beyond the merely academic. Second, they suggest that there is a need to carry out scientific research in the area of self-regulation, using instruments such as the SRL vs ERL instrument in different contexts so as to identify the strengths and areas for improvement of this new model. Results so far indicate that it is of greater utility for identifying important aspects that more traditional instruments do not take fully into account, in particular in relation to different levels of regulation and the distinction between internal and external regulation (Pintrich, 2004; de la Fuente et al., 2017, 2019, 2020b,2022b; Goffena and Horn, 2021; Pachón-Basallo et al., 2021; Tinner et al., 2021).

In addition, in light of the results found, it is important for educational psychology to incorporate external regulation of learning behavior in its vision of effective teaching (Entwistle and Peterson, 2004; Roehrig and Christesen, 2010), since teaching students to regulate their own learning behavior will bring advantages for them inside and outside the classroom. It is to be hoped that external regulation of learning will prompt self-regulating students in their study processes and in turn promote self-regulation in other areas of their lives (Zimmerman and Schunk, 1989; Yerdelen and Sungur, 2019). It is also probable that students who perceive that the regulation of their learning is externally facilitated will have a greater appreciation of the teaching process, which will once again impact their well-being. We hope in future research to explain how that comes about (Goe et al., 2008; de la Fuente, 2017; Putwain and Pescod, 2018; Baherimoghadam et al., 2021; Bakhtiar and Hadwin, 2022).

### Limitations

This research has limitations which should be mentioned. First, there are limitations concerning the sample, which did not have enough participants to make high-level population scale inferences. Second, the initial validation of the instruments used in this research in relation to the internal and external regulation of learning was carried out in the same sample. Consequently, further revalidation studies of the specific instruments should be performed on the categorization of self-regulation vs. hetero regulation. In addition, no account was taken of possible differences arising from age, sex, or other relevant sociodemographic variables and their possible impact on the relationships among the variables considered.

### Future research

Future research should continue to validate the factorial invariance of these relationships in other contexts, such as in organizations, social contexts, in teaching, in the use of ITC, etc. First, the adequacy of the categorization of dimensions of regulation (SRL/ERL-NRL/ENL, DRL/EDL), which might assist in classifying behavioral problems, should itself be confirmed. Second, cross-cultural studies should be performed to gather evidence of the intercultural validity of that categorization and the instruments developed to assess those constructs. In addition, there would be value in future research to determine the weight of each context – distinguishing family, school, and peers – in these predictive analyses so as to determine any discrepancies or similarities between the perceptions that students have. On the path toward those goals, these preliminary results provide empirical support for the proposed General SR vs. ER Theory (de la Fuente, 2021, 2022; de la Fuente et al., 2022a,b).

## Data availability statement

The raw data supporting the conclusions of this article will be made available by the authors, without undue reservation.

## Ethics statement

The studies involving human participants were reviewed and approved by http://www.estres.investigacion-psicopedagogica.org/lib/pdf/CERTIFICADO_COMITE_DE_ETICA_UNAV.pdf. The patients/participants provided their written informed consent to participate in this study.

## Author contributions

MP-B: study design, data analysis, and drafting of the initial report. JF: director of the thesis and R&D project. MG-T: supervision and revision of the writing of the article. JM-V: director of the R&D project. FP-S and MV-M: data collection. All authors contributed to the article and approved the submitted version.

## References

[B1] Aluja-Fabregat A. Blanch A. (2004). Socialized personality, scholastic aptitudes, study habits, and academic achievement: Exploring the link. *Eur. J. Psychol. Assess.* 20 157–165. 10.1027/1015-5759.20.3.157

[B2] Anderson T. Rourke L. Garrison R. Archer W. (2019). Assessing teaching presence in a computer conferencing context. *Online Learn.* 5 1–17. 10.24059/olj.v5i2.1875 33692645

[B3] Arbuckle J. L. (2014). *Amos (Version 23.0) [Computer Program].* Chicago, IL: IBM SPSS.

[B4] Baherimoghadam T. Hamedani S. Mehrabi M. Naseri N. Marzban N. (2021). The effect of learning style and general self-efficacy on satisfaction of e-learning in dental students. *BMC Med. Educ.* 21:463. 10.1186/s12909-021-02903-5 34461883 PMC8405388

[B5] Bakhtiar A. Hadwin A. F. (2022). Motivation from a self-regulated learning perspective: Application to school psychology. *Can. J. Sch. Psychol.* 37 93–116. 10.1177/08295735211054699

[B6] Bandura A. (1986). *Social Foundations of Thought and Action: A Social Cognitive Theory.* Englewood Cliffs, NJ: Prentice-Hall.

[B7] Bandura A. Schunk D. (1981). Cultivating competence, self-efficacy, and intrinsic interest through proximal self-motivation. *J. Pers. Soc. Psychol.* 41 586–598.

[B8] Baumeister R. F. Heatherton T. F. (1996). Self-regulation failure: An overview. *Psychol Inq.* 7 1–15. 10.1207/s15327965pli0701_1

[B9] Biggs J. (2001). *Teaching for Quality Learning at University*, 3rd Edn. Buckingham, UK: Open University Press.

[B10] Biggs J. (2003). *Teaching for Quality Learning at University: What the Student Does*, 2nd Edn. Bristol, PA: SRHE and Open University Press.

[B11] Bobe B. J. Cooper B. J. (2019). The effect of language proficiency on approaches to learning and satisfaction of undergraduate accounting students. *Account. Educ.* 28 149–171. 10.1080/09639284.2017.1396481

[B12] Booth P. Luckett P. Mladenovic R. (1999). The quality of learning in accounting education: The impact of approaches to learning on academic performance. *Account. Educ.* 8 277–300. 10.1080/096392899330801

[B13] Brainerd C. J. Zimmerman B. J. Schunk D. H. (1989). *Self-Regulated Learning and Academic Achievement: Theory, Research, and Practice.* Berlin: Springer.

[B14] Brown J. M. (1998). “Self-regulation and the addictive behaviors,” in *Treating Addictive Behaviors*, Vol. 2 eds Miller W. R. Heather N. (New York, NY: Plenum Press), 61–73. 10.1007/978-1-4899-1934-2_5

[B15] Brown J. M. Miller W. R. Lawendowski L. A. (1999). “The self-regulation questionnaire,” in *Innovations in Clinical Practice: A Source Book*, Vol. 17 eds Vandecreek L. Jackson T. L. (Sarasota, FL: Professional Resource Press), 281–292.

[B16] Campano L. Robledo P. Algorri L. (2017). Análisis del uso de estrategias de aprendizaje cognitivas y metacognitivas en educación secundaria. *Eur. J. Child Dev. Educ. Psychopathol.* 5:97. 10.30552/ejpad.v5i2.51

[B17] Carver C. S. Scheier M. F. (1998). *On the Self-Regulation of Behavior*, 1st Edn. Cambridge, MA: Cambridge University Press. 10.1017/CBO9781139174794

[B18] Caskurlu S. Maeda Y. Richardson J. C. Lv J. (2020). A meta-analysis addressing the relationship between teaching presence and students’ satisfaction and learning. *Comput. Educ.* 157:103966. 10.1016/j.compedu.2020.103966

[B19] Caso-Niebla J. Hernández-Guzmán L. (2007). Variables que inciden en el rendimiento académico de adolescentes mexicanos (Variables that affect the academic performance of Mexican teenagers). *Rev. Latinoam. Psicol.* 39 487–501.

[B20] Cohen M. T. (2012). The importance of self-regulation for college student learning. *Coll. Stud. J.* 46 892–902.

[B21] Corno L. (1994). “Student volition and education: Outcomes, influences, and practices,” in *Self-Regulation of Learning and Performance: Issues and Educational Applications*, eds Schunk D. H. Zimmerman B. J. (Mahwah, NJ: Lawrence Erlbaum Associates), 229–251.

[B22] Dansereau D. F. (1985). *Learning Strategy Research*, Vol. 1. Mahwah, NJ: Lawrence Erlbaum Associates.

[B23] de la Fuente J. (2017). Theory of Self- vs. Externally-regulated learning™: Fundamentals, evidence, and applicability. *Front. Psychol.* 8:1675. 10.3389/fpsyg.2017.01675 29033872 PMC5627139

[B24] de la Fuente J. (2020). *Self-Regulation vs. External Regulation of Learning Scale (SRL vs ERL).* Pamplona: University of Navarra.

[B25] de la Fuente J. (2021). *Theory of Self- vs. Externally-Regulated Behavior: Fundamentals, Evidence, and Applicability.* Pamplona: University of Navarra.

[B26] de la Fuente J. (2022). *Self- vs. External Regulation Behavior Scales.* Pamplona: University of Navarra.

[B27] de la Fuente J. Amate J. González-Torres M. C. Artuch R. García-Torrecillas J. M. Fadda S. (2020a). Effects of levels of self-regulation and regulatory teaching on strategies for coping with academic stress in undergraduate students. *Front. Psychol.* 11:22. 10.3389/fpsyg.2020.00022 32082213 PMC7005059

[B28] de la Fuente J. Sander P. Kauffman D. F. Yilmaz Soylu M. (2020b). Differential effects of self- vs. external-regulation on learning approaches, academic achievement, and satisfaction in undergraduate students. *Front. Psychol.* 11:543884. 10.3389/fpsyg.2020.543884 33117221 PMC7575817

[B29] de la Fuente J. Justicia F. Sander P. Cardelle-Elawar M. (2014). Personal self-regulation and regulatory teaching to predict performance and academic confidence: New evidence for the DEDEPRO Model™. *Electron. J. Res. Educ. Psychol.* 12 597–620. 10.14204/ejrep.34.14031

[B30] de la Fuente J. López M. Zapata L. Solinas G. Fadda S. (2015). “Improving mental health through an online self-assessment and self-help e-utility in university students,” in *Progress in Education*, Vol. 33 ed. Nata R. (Hauppauge, NY: Nova Publisher), 63–76.

[B31] de la Fuente J. Sander P. Garzón-Umerenkova A. Vera-Martínez M. M. Fadda S. Gaetha M. L. (2021b). Self-regulation and regulatory teaching as determinants of academic behavioral confidence and procrastination in undergraduate students. *Front. Psychol.* 12:182. 10.3389/fpsyg.2021.602904 33643135 PMC7902717

[B32] de la Fuente J. Malpica-Chavarria E. A. Garzón-Umerenkova A. Pachón-Basallo M. (2021a). Effect of personal and contextual factors of regulation of academic achievement during adolescence: The role of gender and age. *Int. J. Environ. Res. Public Health* 18:8944. 10.3390/ijerph18178944 34501534 PMC8431230

[B33] de la Fuente J. Martínez-Vicente J. M. Pachón-Basallo M. Peralta-Sánchez F. J. Vera-Martínez M. M. Andrés-Romero M. P. (2022a). Differential predictive effect of self-regulation behavior and the combination of self- vs. external regulation behavior on executive dysfunctions and emotion regulation difficulties, in university students. *Front. Psychol.* 13:876292. 10.3389/fpsyg.2022.876292 35814083 PMC9258503

[B34] de la Fuente J. Martínez-Vicente J. M. Santos F. H. Sander P. Fadda S. Karagiannopoulou A. (2022b). Advances on self-regulation models: A new research agenda through the SR vs ER behavior Theory in different psychology contexts. *Front. Psychol.* 13:861493. 10.3389/fpsyg.2022.861493 PMC9336543 35910968

[B35] de la Fuente J. Martínez-Vicente J. M. Peralta-Sánchez F. J. Garzón-Umerenkova A. Vera M. M. Paoloni P. (2019). Applying the SRL vs. ERL theory to the knowledge of achievement emotions in undergraduate university students. *Front. Psychol.* 10:2070. 10.3389/fpsyg.2019.02070 31620044 PMC6760021

[B36] de la Fuente J. Sander P. Martínez-Vicente J. M. Vera M. Garzón A. Fadda S. (2017). Combined effect of levels in personal self-regulation and regulatory teaching on meta-cognitive, on meta-motivational, and on academic achievement variables in undergraduate students. *Front. Psychol.* 8:232. 10.3389/fpsyg.2017.00232 28280473 PMC5322205

[B37] de la Fuente J. Zapata L. Martínez-Vicente J. M. Cardelle-Elawar M. Sander P. Justicia F. (2012). Regulatory teaching and self-regulated learning in college students: Confirmatory validation study of the IATLP scales. *Electron. J. Res. Educ. Psychol* 10 839–866.

[B38] Dinsmore D. L. Alexander P. A. Loughlin S. M. (2008). Focusing the conceptual lens on metacognition, self-regulation, and self-regulated learning. *Educ. Psychol. Rev.* 20 391–409. 10.1007/s10648-008-9083-6

[B39] Duckworth A. L. Tsukayama E. Kirby T. A. (2013). Is it really self-control? Examining the predictive power of the delay of gratification task. *Pers. Soc. Psychol. Bull.* 39 843–855. 10.1177/0146167213482589 23813422 PMC3794428

[B40] Enders C. K. Bandalos D. L. (2001). The relative performance of full information maximum likelihood estimation for missing data in structural equation models. *Struct. Equ. Modeling.* 8 430–457. 10.1207/S15328007SEM0803_5 33486653

[B41] Entwistle N. J. Peterson E. R. (2004). Conceptions of learning and knowledge in higher education: Relationships with study behavior and influences of learning environments. *Int. J. Educ. Res.* 41 407–428. 10.1016/j.ijer.2005.08.009

[B42] Ertmer P. A. Newby T. J. (1996). The expert learner: Strategic, self-regulated, and reflective. *Instr. Sci.* 24 1–24. 10.1007/BF00156001

[B43] Flavell J. H. (1987). “Speculations about the nature and development of metacognition,” in *Metacognition, Motivation, and Understanding*, eds Weinert F. E. Kluwe R. (Mahwah, NJ: Lawrence Erlbaum Associates), 21–29. 10.1016/s0013-7006(04)95472-3

[B44] Gambo Y. Shakir M. Z. (2021). Review on self-regulated learning in smart learning environment. *Smart Learn. Environ.* 8:12. 10.1186/s40561-021-00157-8

[B45] Garzón-Umerenkova A. de la Fuente J. Amate J. Paoloni P. V. Fadda S. Pérez J. F. (2018). A linear empirical model of self-regulation on flourishing, health, procrastination, and achievement, among university students. *Front. Psychol.* 9:536. 10.3389/fpsyg.2018.00536 29706922 PMC5909179

[B46] Goe L. Bell C. Little O. (2008). *Approaches to Evaluating Teacher Effectiveness: A Research Synthesis.* Washington, DC: National Comprehensive Center for Teacher Quality.

[B47] Goffena J. D. Horn T. S. (2021). The relationship between coach behavior and athlete self-regulated learning. *Int. J. Sports Sci. Coach.* 16 3–15. 10.1177/1747954120951903

[B48] Gonzáles-Torres M. C. Torrano F. (2008). “Methods and instruments for measuring self-regulated learning,” in *Handbook of Instructional Resources & Applications*, eds Valle A. Núñez J. C. (Hauppauge, NY: Nova Science Publishers).

[B49] González-Pienda J. A. Núñez Pérez J. C. García Rodríguez M. (1998). “Estrategias de aprendizaje (Learning Strategies),” in *Dificultades del Aprendizaje Escolar (Difficulties in Learning at School)*, eds González-Pienda J. A. Núñez Pérez J. C. (Madrid: Pirámide), 127–156.

[B50] González-Torres M. C. (1997). *La Motivación Académica. Sus Determinantes y Pautas de Intervención (Academic Motivation. Its Determinants and Guidelines for Intervention).* Pamplona: Eunsa.

[B51] González-Torres M. C. Tourón J. (1992). *Autoconcepto y Rendimiento Escolar: Sus Implicaciones En La Motivación y En La Autorregulación del Aprendizaje (Self-perception and Academic Performance: Its implications for the motivation and self-regulation of learning).* Pamplona: Eunsa.

[B52] Hair J. William B. Barry B. Anderson R. (2010). *Multivariate Data Analysis*, 7th Edn. Hoboken, NJ: Prentice Hall.

[B53] Hammerness K. M. Darling-Hammond L. Bransford J. (2005). “How teachers learn and develop,” in *Preparing Teachers for a Changing World: What Teachers Should Learn and Be Able to Do*, eds Darling-Hammond L. Bransford J. (San Francisco, CA: Jossey-Bass), 358–389.

[B54] Hennessy D. A. Doğan E. B. Rothengatter T. Steg L. Delhomme P. (eds) (2011). “Self-regulation and driving behavior,” in *Traffic Psychology an International Perspective*, eds Hennessy D. A. Doðan E. B. Rothengatter T. Steg L. Delhomme P. (Hauppauge, NY: Nova Science Publishers), 129–143.

[B55] Hernández P. García L. A. (1995). *Cuestionario de Estrategias de Control en el Studio (Study Control Strategies Questionnaire) (ECE).* San Cristóbal de La Laguna: University of La Laguna.

[B56] Holdfor D. Patkar A. (2003). Identification of the service quality dimensions of pharmaceutical education. *Am. J. Pharm. Educ.* 67 849–859.

[B57] Hu L. Bentler P. M. (1999). Cutoff criteria for fit indexes in covariance structure analysis: Conventional criteria versus new alternatives. *Struct. Equ. Modeling.* 6 1–55. 10.1080/10705519909540118

[B58] Hwang G.-J. Wang S.-Y. Lai C.-L. (2021). Effects of a social regulation-based online learning framework on students’ learning achievements and behaviors in mathematics. *Comput. Educ.* 160:104031. 10.1016/j.compedu.2020.104031

[B59] IBM Corp (2019). *IBM SPSS Statistics for Windows, Version 26.0.* Armonk, NY: IBM Corp.

[B60] Inglés C. Martínez-González A. García-Fernández J. (2013). Conducta prosocial y estrategias de aprendizaje en una muestra de estudiantes españoles de educación secundaria obligatoria (Prosocial behavior and learning strategies in a sample of Spanish students undergoing compulsory secondary education). *Eur. J. Educ. Psychol.* 6 33–53.

[B61] Jöreskog K. G. Sörbom D. (1998). *LISREL 8: Structural Equation Modeling With the SIMPLIS Command Language (4. print. (With Foreword and Computer Exercises)).* Mahwah, NJ: Erlbaum.

[B62] Kanfer F. H. (1970). “Self-regulation: Research, issues, and speculations,” in *Behavior Modification in Clinical Psychology*, eds Neuringer C. Michael J. L. (New York, NY: Appleton-Century-Crofts), 178–220.

[B63] Kanfer F. H. (1986). “Implications of a self-regulation model of therapy for treatment of addictive behaviors,” in *Treating Addictive Behaviors*, eds Miller W. R. Heather N. (Berlin: Springer), 29–47. 10.1007/978-1-4613-2191-0_2

[B64] Kapoor H. Inamdar V. Kaufman J. C. (2021). I didn’t have time! a qualitative exploration of misbehaviors in academic contexts. *J. Acad. Ethics* 20 191–208. 10.1007/s10805-021-09407-3

[B65] Kapoor H. Kaufman J. C. (2020). Are cheaters common or creative?: Person-situation interactions of resistance in learning contexts. *J. Acad. Ethics* 19 157–174. 10.1007/s10805-020-09379-w

[B66] Karniol R. Miller D. T. (1983). Why not wait?: A cognitive model of self-imposed delay termination. *J. Pers. Soc. Psychol.* 45 935–942. 10.1037/0022-3514.45.4.935

[B67] Keith T. Z. (2019). *Multiple Regression and Beyond: An Introduction to Multiple Regression and Structural Equation Modeling*, 3rd Edn. Oxfordshire: Routledge. 10.4324/9781315162348

[B68] Kline R. B. (2016). *Principles and Practice of Structural Equation Modeling*, 4th Edn. New York, NY: The Guilford Press.

[B69] Koenig E. Guertler K. (2021). One size does not fit all: Individuality and perceptions of improvement and satisfaction among TE students. *Engl. Teach. Learn.* 45 303–324. 10.1007/s42321-021-00076-4

[B70] Kuhl J. (2000). “A functional-design approach to motivation and self-regulation,” in *Handbook of Self-Regulation*, eds Boekaerts M. Pintrich P. Zeidner M. (Cambridge, MA: Academic Press), 111–169.

[B71] Lanza D. Sánchez V. (2014). Apoyo parental y su incidencia en el desarrollo de estrategias de aprendizaje en educación secundaria: Un estudio exploratorio (Parental support and its impact on the implementation of learning strategies in secondary education: An exploratory study). *Int. J. Dev. Educ. Psychol.* 2 489–499.

[B72] Lohr S. L. (2010). *Sampling: Design and Analysis*, 2nd Edn. Pacific Grove, CA: Brooks/Cole.

[B73] Mardia K. V. (1970). Measures of multivariate skewness and kurtosis with applications. *Biometrika* 57 519–530. 10.1093/biomet/57.3.519

[B74] Martínez-Fernández R. (2007). Concepción de aprendizaje y estrategias metacognitivas en estudiantes universitarios de psicología (conception of learning and metacognitive strategies in university psychology students). *An. Psicol.* 23 7–16.

[B75] McCombs B. L. Marzano R. J. (1990). Putting the self in self-regulated learning: The self as agent in integrating will and skill. *Educ. Psychol.* 25 51–69. 10.1207/s15326985ep2501_5 33486653

[B76] Miller W. R. Brown J. M. (1991). “Self-regulation as a conceptual basis for the prevention and treatment of addictive behaviours,” in *Self-Control and the Addictive Behaviours*, eds Heather N. Miller W. R. Greeley J. (New York, NY: Maxwell Macmillan), 3–79. 10.1016/j.addbeh.2018.07.014

[B77] Miller W. R. Rollnick S. (2013). *Motivational Interviewing: Helping People Change*, 3rd Edn. New York, NY: Guilford Press.

[B78] Monereo C. (2007). Towards a new paradigm of strategic learning: The role of social mediation, self and emotions. *Electron. J. Res. Educ. Psychol.* 5 497–534.

[B79] Muntada M. (2013). Personality, procrastination and cheating in students from different university degree programs. *Electron. J. Res. Educ. Psychol.* 11 451–472. 10.14204/ejrep.30.13030

[B80] Navarro-Patón R. Mecías-Calvo M. Eirín-Nemiña R. Arufe-Giráldez V. (2022). Disruptive behaviors in physical education: A matched study of social skills and sport practice in a region of Spain. *Int. J. Environ. Res. Public Health* 19:1166. 10.3390/ijerph19031166 35162189 PMC8834815

[B81] Nilson L. B. (2013). *Creating Self-Regulated Learners: Strategies to Strengthen Students’ Self-Awareness and Learning Skills.* Richmond, VA: Sterling.

[B82] Nisbet J. Shucksmith J. (2017). *Learning Strategies*, 1st Edn. Oxfordshire: Routledge. 10.4324/9781315188652

[B83] Pachón-Basallo M. de la Fuente J. Gonzáles-Torres M. C. (2021). Regulation/Non-regulation/dys-regulation of health behavior, psychological reactance, and health of university undergraduate students. *Int. J. Environ. Res. Public Health* 18:3793. 10.3390/ijerph18073793 33916478 PMC8038604

[B84] Panadero E. (2017). A review of self-regulated learning: Six models and four directions for research. *Front. Psychol.* 8:422. 10.3389/fpsyg.2017.00422 28503157 PMC5408091

[B85] Pérez Posada D. C. Londoño-Vásquez D. A. (2015). La influencia de la familia en el desempeño académico de los y las adolescentes del grado sexto en tres instituciones de Antioquia. The family influence in sixth grade adolescents’ academic performance in three Antioquia’s institutions. *Psicoespacios* 9:215. 10.25057/21452776.359

[B86] Pichardo M. C. Cano F. Garzón-Umerenkova A. de la Fuente J. Peralta-Sánchez F. J. Amate-Romera J. (2018). Self-regulation questionnaire (SRQ) in Spanish adolescents: Factor structure and rasch analysis. *Front. Psychol.* 9:1370. 10.3389/fpsyg.2018.01370 30147667 PMC6095962

[B87] Pinho A. S. Molleman L. Braams B. R. van den Bos W. (2021). Majority and popularity effects on norm formation in adolescence. *Sci. Rep.* 11:12884. 10.1038/s41598-021-92482-8 34145360 PMC8213745

[B88] Pintrich P. R. (2000). “The role of goal orientation in self-regulated learning,” in *Handbook of Self-Regulation*, eds Boekaerts M. Pintrich P. Zeidner M. (Cambridge, MA: Academic Press), 451–502.

[B89] Pintrich P. R. (2004). A conceptual framework for assessing motivation and self-regulated learning in college students. *Educ. Psychol. Rev.* 16 385–407.

[B90] Popa D. (2015). The relationship between self-regulation, motivation and performance at secondary school students. *Proc. Soc. Behav. Sci.* 191 2549–2553. 10.1016/j.sbspro.2015.04.410

[B91] Putwain D. W. Pescod M. (2018). Is reducing uncertain control the key to successful test anxiety intervention for secondary school students? Findings from a randomized control trial. *Sch. Psychol. Q.* 33 283–292. 10.1037/spq0000228 29094957

[B92] Puustinen M. Pulkkinen L. (2001). Models of self-regulated learning: A review. *Scand. J. Educ. Res.* 45 269–286. 10.1080/00313830120074206

[B93] Quero-Virla M. (2010). Confiabilidad y coeficiente alpha de cronbach. *Telos* 12 248–252.

[B94] Roces C. González-Torres M. C. (1998). “Capacidad de autorregulación del proceso de aprendizaje (Capacity for self-regulation of the learning process),” in *Dificultades del Aprendizaje Escolar (Difficulties in Learning at School)*, eds González-Pienda J. A. Núñez Pérez J. C. (Madrid: Pirámide), 239–261.

[B95] Rodríguez G. (2009). *Motivación, Estrategias de Aprendizaje y Rendimiento Académico en Estudiantes de ESO.* A Coruña: University of a Coruña.

[B96] Roehrig A. D. Christesen E. (2010). “Development and use of a tool for evaluating teacher effectiveness in grades K-12,” in *Innovative Assessment for the 21st Century*, eds Shute V. J. Becker B. J. (Berlin: Springer), 207–228. 10.1007/978-1-4419-6530-1_12

[B97] Roth A. Ogrin S. Schmitz B. (2016). Assessing self-regulated learning in higher education: A systematic literature review of self-report instruments. *Educ. Assess. Eval. Account.* 28 225–250. 10.1007/s11092-015-9229-2

[B98] Ryan R. M. Deci E. L. (2017). *Self-Determination Theory: Basic Psychological Needs in Motivation, Development, and Wellness.* New York, NY: The Guilford Press. 10.1521/978.14625/28806

[B99] Schuitema J. Peetsma T. Van Der Veen I. (2012). Self-regulated learning and students’ perceptions of innovative and traditional learning environments: A longitudinal study in secondary education. *Educ. Stud.* 38 397–413. 10.1080/03055698.2011.643105

[B100] Schunk D. H. (2005). Inherent details of self-regulated learning include student perception. *Educ. Psychol.* 30 213–216. 10.1207/s15326985ep3004_7

[B101] Smale W. T. Hutcheson R. Russo C. J. (2021). Cell phones, student rights, and school safety: Finding the right balance. *Can. J. Educ. Adm. Policy* 195 49–64. 10.7202/1075672ar 33270396

[B102] Tabachnick B. G. Fidell L. S. (2001). *Using Multivariate Statistics*, 4th Edn. Boston, MA: Allyn and Bacon.

[B103] The World Bank. (2021). *). School Life Expectancy. Global Innovation Index.* Washington, DC: The World Bank.

[B104] Tinner L. Caldwell D. Campbell R. (2021). Community mobilisation approaches to preventing and reducing adolescent multiple risk behaviour: A realist review protocol. *Syst. Rev.* 10:147. 10.1186/s13643-021-01696-4 33980307 PMC8117311

[B105] Torrano F. González-Torres M. C. (2004). Self-regulated learning: Current and future directions. *Electron. J. Res. Educ. Psychol.* 2 1–34. 10.25115/ejrep.3.120

[B106] Valle A. Núñez J. C. Cabanach R. G. Rodríguez S. González-Pienda J. A. Rosario P. (2007). Metas académicas y estrategias motivacionales de Autoprotección (Academic goals and Self-Protective Motivational Strategies). *Electron. J. Res. Educ. Psychol.* 5 617–632.

[B107] Watson-Brown N. Senserrick T. Freeman J. Davey J. Scott-Parker B. (2021). Self-regulation differences across learner and probationary drivers: The impact on risky driving behaviours. *Accid. Anal. Prev.* 154:106064. 10.1016/j.aap.2021.106064 33721731

[B108] Weinstein C. E. Mayer R. F. (1986). “The teaching of learning Strategies,” in *Handbook of Research on Teaching*, ed. Wittrock M. C. (New York, NY: McMillan), 315–327.

[B109] Weston R. Gore P. A. (2006). A brief guide to structural equation modeling. *Counsel. Psychol.* 34 719–751. 10.1177/0011000006286345

[B110] Wu Y.-C. Hsieh L.-F. Lu J.-J. (2015). What’s the relationship between learning satisfaction and continuing learning intention? *Proc. Soc. Behav. Sci.* 191 2849–2854. 10.1016/j.sbspro.2015.04.148

[B111] Yerdelen S. Sungur S. (2019). Multilevel investigation of students’ self-regulation processes in learning science: Classroom learning environment and teacher effectiveness. *Int. J. Sci. Math. Educ.* 17 89–110. 10.1007/s10763-018-9921-z

[B112] Young A. Fry J. (2012). Metacognitive awareness and academic achievement in college students. *J. Scholarsh. Teach. Learn.* 8 1–10.

[B113] Zimmerman B. J. (1988). “A social cognitive view of self-regulated academic learning,” in *Paper Presented at the Annual Meeting of the American Educational Research Association*, New Orleans, LA. 10.1006/ceps.1999.1002

[B114] Zimmerman B. J. (1989). “Self-regulated learning and academic achievement: Theory, research, and practice,” in *Self-Regulated learning and Academic Achievement: An Overview and Analysis*, eds Zimmerman B. J. Schunk D. (Oxfordshire: Taylor&Francis), 1–25.

[B115] Zimmerman B. J. (2000). “Attaining self-regulation: A social cognitive perspective,” in *Handbook of Self-Regulation*, eds Boekaerts M. Pintrich P. Zeidner M. (San Diego, CA: Academic Press), 13–39.

[B116] Zimmerman B. J. (2002). Becoming a self-regulated learner: An overview. *Theory Pract.* 41 64–70. 10.1207/s15430421tip4102_2

[B117] Zimmerman B. J. (2008). Investigating self-regulation and motivation: Historical background, methodological developments, and future prospects. *Am. Educ. Res. J.* 45 166–183. 10.3102/0002831207312909

[B118] Zimmerman B. J. (2015). “Self-regulated learning: Theories, measures, and outcomes,” in *International Encyclopedia of the Social & Behavioral Sciences*, ed. Wright J. D. (Oxford: Elsevier), 541–546. 10.1016/B978-0-08-097086-8.26060-1

[B119] Zimmerman B. J. Labuhn A. S. (2012). “Self-regulation of learning: Process approaches to personal development,” in *APA Educational Psychology Handbook, Vol 1: Theories, Constructs, and Critical Issues*, eds Harris K. R. Graham S. Urdan T. McCormick C. B. Sinatra G. M. Sweller J. (Washington, DC: American Psychological Association), 399–425. 10.1037/13273-014

[B120] Zimmerman B. J. Schunk D. (1989). *Self-Regulated Learning and Academic Achievement: An Overview and Analysis.* Oxfordshire: Taylor&Francis.

[B121] Zimmerman J. Schunk D. DiBeneddetto M. (2017). “The role of self-efficacy and related beliefs in self-regulation of learning and performance,” in *Handbook of Competence and Motivation: Theory and Application*, 2nd Edn, eds Elliot A. Dweck C. Yeager D. (New York, NY: The Guilford Press), 313–333.

